# Thyroid hormone deficiency induces endolymphatic hydrops: neurological and histopathological evidence from an experimental rat model

**DOI:** 10.3389/fneur.2026.1774666

**Published:** 2026-02-06

**Authors:** Gizem Meral Kantarci, Serap Sahin Onder, Elif Gelenli Dolanbay, Mehmet Surmeli, Eray Metin Guler, Hacer Inan Gungor, Asli Sahin Yilmaz

**Affiliations:** 1 Umraniye Egitim Ve Arastirma Hastanesi, Istanbul, Türkiye; 2 Acibadem Atasehir Hospital, Istanbul, Türkiye; 3 Istanbul Medeniyet Universitesi, Istanbul, Türkiye; 4 Istanbul Haydarpasa Numune Egitim ve Arastirma Hastanesi, Istanbul, Türkiye

**Keywords:** endolymphatic hydrops, hypothyroidism, Ménière’s disease, neuro-otology, thyroidectomy, vestibular disorders

## Abstract

**Background:**

Vestibular disorders, particularly Ménière’s disease, represent significant neurological conditions affecting balance, spatial orientation, and quality of life. While endolymphatic hydrops is recognized as the pathological hallmark of Ménière’s disease, the relationship between thyroid dysfunction and vestibular pathology remains incompletely understood. Clinical observations suggest associations between hypothyroidism and vestibular symptoms, yet experimental evidence demonstrating causality is lacking.

**Objective:**

To investigate whether surgically-induced hypothyroidism causes endolymphatic hydrops development in an experimental rat model and to characterize the histopathological changes in vestibular structures.

**Methods:**

Twelve male Wistar albino rats were randomly allocated to total thyroidectomy (*n* = 4), sham surgery (*n* = 4), and control (*n* = 4) groups. Thyroid function was assessed via serum thyroid-stimulating hormone (TSH) and thyroxine (T4) measurements at baseline and postoperative day 15. Animals were euthanized at 28 days post-surgery for comprehensive histopathological examination of temporal bones. Endolymphatic hydrops was evaluated using standardized semiquantitative scoring systems for Reissner’s membrane, vestibular structures, stria vascularis, and utricular macula.

**Results:**

Thyroidectomized rats developed significant biochemical hypothyroidism with elevated TSH (mean difference: 0.378 μIU/mL, 95% CI: 0.183–0.573, *p* = 0.021) and decreased T4 levels (mean difference: −2.357 pmol/L, 95% CI: −3.521 to −1.193, *p* = 0.021). Histopathological examination revealed universal development of endolymphatic hydrops in all thyroidectomized animals (100% vs. 0% in controls, *p* < 0.001), affecting Reissner’s membrane, vestibular apparatus, stria vascularis, and utricular macula with varying severity.

**Conclusion:**

This study provides the first direct experimental evidence that thyroid hormone deficiency induces endolymphatic hydrops in all examined vestibular structures. These findings establish a mechanistic link between hypothyroidism and vestibular pathology, with important implications for the neurological evaluation and management of patients presenting with vestibular symptoms and comorbid thyroid dysfunction.

## Introduction

The vestibular system, a critical component of the neurological network controlling spatial orientation and balance, relies on precise regulation of inner ear fluid homeostasis (1). Vestibular disorders impose substantial neurological burden, with Ménière’s disease representing one of the most debilitating conditions characterized by episodic vertigo, fluctuating sensorineural hearing loss, tinnitus, and aural fullness (2). The temporal patterns of vertiginous episodes vary considerably, with inter-attack intervals ranging from days to months (3). While symptom severity diminishes progressively in 60%–80% of patients over 2–8 years, this improvement typically occurs alongside permanent sensorineural hearing loss and persistent tinnitus (4). Recent diagnostic criteria established by the Bárány Society Classification Committee have simplified the framework to probable and definite categories of Ménière’s disease (5).

Endolymphatic hydrops, characterized by expansion of the scala media through bulging of Reissner’s membrane into the scala vestibuli, constitutes the fundamental pathological substrate of Ménière’s disease (6). However, it is important to note that endolymphatic hydrops represents a histopathological finding rather than a clinical diagnosis; Ménière’s disease itself is a multifactorial clinical syndrome whose pathogenesis extends beyond hydrops alone and may involve genetic susceptibility, autoimmune mechanisms, and vascular factors (6). Despite well-established clinical associations, the precise molecular and cellular mechanisms underlying hydrops development remain elusive, necessitating experimental animal models to elucidate the pathophysiological basis of this disorder (7). Since Kimura and Schuknecht first demonstrated that surgical obliteration of the endolymphatic duct in guinea pigs produces hydrops (8), numerous experimental approaches have been developed to model this condition.

From a neurological perspective, the vestibular system’s integration with central nervous system pathways controlling gaze stabilization, postural control, and spatial cognition underscores the importance of understanding peripheral vestibular pathology (9). The thyroid gland exerts widespread influence on neurological function through the hypothalamic–pituitary-thyroid axis, affecting both systemic and organ-specific metabolic processes (10). Hypothyroidism, characterized by insufficient circulating thyroid hormone levels, represents one of the most prevalent endocrine disorders globally, with autoimmune thyroiditis (Hashimoto’s thyroiditis) constituting the predominant etiology in iodine-sufficient regions.

Several biological pathways may explain the potential link between thyroid hormone deficiency and inner ear dysfunction. Thyroid hormone receptors (TRα and TRβ) are expressed in cochlear and vestibular structures, including spiral ganglion neurons, hair cells, and the stria vascularis (11). Thyroid hormones regulate Na, K-ATPase expression, an enzyme essential for maintaining endocochlear potential and endolymph homeostasis in the stria vascularis (12). Additionally, thyroid hormones modulate the expression of potassium channels (KCNQ4, KCNJ10) critical for outer hair cell function and endocochlear potential generation (13). Recent meta-analyses have confirmed an increased risk of sensorineural hearing loss in patients with thyroid dysfunction (14). Despite these theoretical frameworks, direct experimental evidence demonstrating that hypothyroidism causes endolymphatic hydrops has been lacking.

Emerging clinical evidence suggests potential associations between thyroid dysfunction and vestibular pathology. Although the nature of this relationship remains to be experimentally established. Santosh and Rao (15) reported that thyroid hormone concentrations were correlated with severity of vertigo, tinnitus, hearing loss, and aural fullness in Ménière’s disease, and observed symptom improvement following levothyroxine supplementation in hypothyroid individuals. While these clinical observations suggest that vestibular symptoms may be linked to thyroid hormone status, whether this relationship is causal or represents a shared underlying pathophysiology remains unclear. Recent investigations have similarly reported associations between hypothyroidism and vestibular dysfunction, including increased prevalence of gait instability and vestibular symptoms in hypothyroid patients compared to euthyroid controls (16).

Particularly noteworthy are recent findings in pediatric populations demonstrating that children with Hashimoto’s thyroiditis exhibit significant vestibular dysfunction on video head impulse testing and vestibular evoked myogenic potential testing despite being clinically asymptomatic and biochemically euthyroid (17). These electrophysiological abnormalities suggest that thyroid-related processes may induce structural or functional changes in the vestibular system that precede overt clinical symptoms. Such subclinical dysfunction could potentially reflect early histopathological changes, including endolymphatic hydrops, which may only become clinically manifest at more advanced stages. These clinical observations underscore the need for controlled experimental studies to determine whether thyroid dysfunction can directly cause inner ear structural pathology. However, the cross-sectional nature of these clinical studies precludes determination of whether thyroid dysfunction directly causes vestibular impairment or whether both conditions share common predisposing factors.

Despite accumulating clinical evidence suggesting associations between hypothyroidism and endolymphatic hydrops, the histopathological changes governing this relationship remain largely unexplored through controlled experimental investigation. Critically, existing clinical and epidemiological studies are limited by their observational design, which can demonstrate correlation but cannot establish causation. The absence of direct experimental evidence represents a significant gap in our understanding of the thyroid-vestibular axis.

From a clinical perspective, the management of Ménière’s disease remains challenging despite various pharmacological and interventional approaches. Current treatments, including betahistine and intratympanic steroids, show variable efficacy in controlling vertigo attacks, highlighting the need for better understanding of disease pathophysiology and identification of modifiable risk factors (18). If hypothyroidism contributes to endolymphatic hydrops development, thyroid hormone optimization could represent a novel therapeutic target for vestibular symptom management.

Total thyroidectomy was selected as the method for inducing hypothyroidism because it provides rapid, complete, and reproducible thyroid hormone depletion, allowing precise temporal control over the onset of hypothyroidism. While pharmacological models (e.g., propylthiouracil, methimazole) offer alternatives, they may have direct ototoxic effects independent of thyroid status. Although surgical induction may introduce potential confounders such as transient surgical stress and risk of parathyroid disruption, these were controlled through sham-operated animals and calcium supplementation, respectively. Therefore, this study aimed to examine whether surgically-induced hypothyroidism produces endolymphatic hydrops at the histopathological level using an experimental rat model. By employing a controlled experimental design with thyroidectomy, sham surgery, and untreated control groups, we sought to determine whether a causal relationship exists between thyroid hormone deficiency and vestibular pathology, thereby providing mechanistic insights that cannot be obtained from observational clinical studies alone.

## Materials and methods

### Ethical approval and study design

All experimental procedures were conducted at the Experimental Application and Research Center in strict accordance with institutional guidelines for animal care and use. The study protocol received approval from the Local Ethics Committee for Animal Experiments (Ethics Committee Approval No: 2021/26, Date: 22.02.2021). Histopathological analyses were performed at the Department of Histology and Embryology, while biochemical evaluations were conducted at the Department of Medical Biochemistry.

### Animal groups and randomization

Twelve male Wistar albino rats (body weight 250–300 g, age 12–14 weeks) were randomly allocated into three experimental groups using a computer-generated randomization sequence (*n* = 4 per group). Sample size was determined using the resource equation approach, which is recommended for exploratory animal studies when prior data for conventional power analysis are unavailable (19). With bilateral temporal bones analyzed independently (8 observations per group), the error degrees of freedom (DF = 21) falls within the acceptable range of 10–20 recommended by this approach. Allocation concealment was ensured by having an independent investigator, who was not involved in surgical procedures or outcome assessment, perform the group assignments using sequentially numbered opaque sealed envelopes.

#### Group 1 (thyroidectomy group)

Animals underwent total thyroidectomy through a longitudinal midline incision on the ventral neck surface. Following tracheal exposure, thyroid lobes were identified bilaterally and completely excised with meticulous attention to preserve the recurrent laryngeal nerves and parathyroid glands.

#### Group 2 (sham surgery group)

Animals received identical surgical procedures as the thyroidectomy group, including midline neck incision, tracheal exposure, and bilateral thyroid lobes visualization, but without glandular excision. This design controlled for surgical stress and tissue manipulation effects.

#### Group 3 (control group)

Animals received no surgical intervention and served as untreated controls to establish baseline histopathological parameters.

The inclusion of both sham-operated and untreated control groups served distinct purposes: the sham surgery group controlled for potential effects of surgical stress, anesthesia, and tissue manipulation on inner ear structures, while the untreated control group established baseline histopathological parameters. All three groups were analyzed separately without pooling to verify that surgical intervention alone did not induce vestibular pathology.

### Surgical procedures

All surgical procedures were performed by a single experienced surgeon under sterile conditions to minimize inter-operator variability. Animals were anesthetized using a combination of ketamine hydrochloride (50 mg/kg body weight) and xylazine hydrochloride (10 mg/kg body weight) administered via intraperitoneal injection. Anesthetic depth was monitored by assessing pedal withdrawal reflex and respiratory rate throughout the procedure. Following adequate anesthesia confirmation, a 2-cm longitudinal midline incision was made on the anterior neck. Skin and subcutaneous tissues were carefully dissected, and the infrahyoid muscles were retracted laterally to expose both thyroid lobes adjacent to the trachea.

Complete total thyroidectomy was performed with meticulous preservation of the recurrent laryngeal nerves to prevent vocal cord paralysis. Special attention was paid to preserve parathyroid glands to minimize postoperative hypocalcemia. Following thyroid removal or sham manipulation, the incision was closed in layers using absorbable sutures. Postoperatively, thyroidectomized animals received 1% calcium chloride (CaCl₂) supplementation in drinking water to prevent hypocalcemic complications. All animals were housed individually in standard laboratory cages under controlled environmental conditions (12-h light/dark cycle, temperature 22 °C ± 2 °C, humidity 50%–60%) with ad libitum access to food and water.

### Serum thyroid function assessment

To confirm biochemical hypothyroidism development, serum thyroid-stimulating hormone (TSH) and free thyroxine (T4) concentrations were measured at two timepoints: baseline (preoperative) and postoperative day 15. Postoperative day 15 was selected for thyroid function assessment based on experimental evidence indicating that stable biochemical hypothyroidism is typically established within 10–14 days following total thyroidectomy in rodent models, allowing adequate equilibration of the hypothalamic–pituitary–thyroid axis (20). For blood sample collection on postoperative day 15, animals were briefly anesthetized with 3%–5% isoflurane (delivered via inhalation chamber) for induction, followed by maintenance at 2%–3% isoflurane during the sampling procedure. Isoflurane was chosen for blood sampling due to its rapid onset and recovery, minimizing stress and allowing quicker return to normal activity. Blood samples (approximately 0.5 mL) were obtained from the jugular vein. Samples were collected in serum separator tubes, allowed to clot for 30 min at room temperature, and centrifuged at 3000 rpm for 10 min. Serum was separated and stored at −80 °C until analysis. TSH and free T4 levels were measured using enzyme-linked immunosorbent assay (ELISA) kits (Elabscience®, Houston, TX, United States) according to manufacturer’s protocols. Within-group comparisons (baseline vs. postoperative day 15) and between-group comparisons at postoperative day 15 were performed.

### Euthanasia protocol

Animals were observed for 28 days following surgery to allow adequate time for endolymphatic hydrops development. This timeframe was selected based on previous experimental studies demonstrating that 4 weeks represents an optimal period for hydrops formation following various interventions (21, 22). A single terminal time point was chosen for this initial exploratory study to first establish whether hypothyroidism induces hydrops before characterizing its temporal progression. At postoperative day 28, animals were euthanized following the AVMA Guidelines for the Euthanasia of Animals (2020). Deep anesthesia was induced using an overdose of ketamine hydrochloride (100 mg/kg) and xylazine hydrochloride (15 mg/kg) administered via intraperitoneal injection. Adequate depth of anesthesia was confirmed by the absence of pedal withdrawal reflex and corneal reflex prior to proceeding with transcardial perfusion. Animals were then perfused transcardially with 0.9% saline followed by 4% paraformaldehyde in phosphate-buffered saline (pH 7.4) for tissue fixation. Death was confirmed by the cessation of cardiac activity and absence of brainstem reflexes following perfusion.

### Tissue harvesting and fixation protocol

Following perfusion, bilateral temporal bones were carefully excised using microsurgical techniques under stereomicroscopic guidance. The harvested specimens were immersed in 10% neutral buffered formalin solution for 48 h for secondary fixation and preservation. Subsequently, temporal bones underwent decalcification in 10% ethylenediaminetetraacetic acid (EDTA) solution for 4–6 weeks with solution changes every 3 days. Decalcification endpoint was confirmed by radiographic examination and needle penetration testing.

### Histopathological processing

Following complete decalcification, temporal bones were processed through graded alcohol series for dehydration, cleared in xylene, and embedded in paraffin wax. Serial sections (5 μm thickness) were cut using a rotary microtome, with every 20th section (at 100 μm intervals) systematically selected throughout the entire cochlea and vestibular apparatus. Only sections meeting predetermined quality criteria (intact tissue architecture, absence of processing artifacts, and presence of target structures) were included for analysis and mounted on poly-L-lysine-coated slides for hematoxylin and eosin (H&E) staining.

Thyroid tissue specimens obtained from the thyroidectomy group underwent identical processing and H&E staining to confirm complete thyroid tissue removal and verify the absence of residual thyroid parenchyma that might produce hormones.

### Microscopic evaluation and Semiquantitative scoring

Histopathological assessment was conducted by two independent observers blinded to experimental groups using light microscopy (Olympus BX53; Olympus Corporation, Tokyo, Japan). Bilateral temporal bones from each animal were analyzed independently rather than averaged. A minimum of 10 sections from each temporal bone were examined, yielding 40 evaluable sections per experimental group (4 animals × 2 temporal bones × 5 sections per bone). Discrepancies between observers were resolved through consensus review. Temporal bone sections were systematically examined for evidence of endolymphatic hydrops, with particular focus on four anatomical regions: Reissner’s membrane, vestibular apparatus (utricle and saccule), stria vascularis, and utricular macula (Table 1). Reissner’s membrane hydrops was designated as the primary outcome, as it represents the most direct histopathological indicator of endolymphatic hydrops. Vestibular apparatus hydrops, stria vascularis pathology, and utricular macula changes were considered secondary outcomes.

**Table 1:** Semiquantitative scoring systems for inner ear histopathological assessment.

Structure	Score/stage	Criteria
Reissner’s membrane	0	No hydrops
	1	Mild hydrops
	2	Moderate hydrops
	3	Severe hydrops
Stria vascularis	0	No contraction
	1	Mild contraction
	2	Moderate contraction
	3	Severe contraction
Vestibular apparatus	0	Normal anatomy: saccule smaller than utricle, no wall connection
	1	Saccule equal to or larger than utricle, no wall connection
	2	Both structures enlarged and converged with minimal separation
	3	Complete adjacency with rupture

#### Reissner’s membrane evaluation

Hydrops severity was assessed using the semiquantitative scoring system described by Ruding et al. (23), which grades distension from 0 (no hydrops: normal position of Reissner’s membrane) to 3 (severe hydrops: Reissner’s membrane reaching the lateral wall of the cochlea with possible rupture). Intermediate grades include mild hydrops (grade 1: slight displacement toward scala vestibuli) and moderate hydrops (grade 2: marked displacement with significant narrowing of scala vestibuli).

#### Stria vascularis assessment

Pathological changes were evaluated using a four-point scoring system based on intermediate cell contraction (24): grade 0 (no contraction: normal morphology), grade 1 (mild contraction: slight reduction in intermediate cell size), grade 2 (moderate contraction: obvious shrinkage with preserved cell structure), and grade 3 (severe contraction: marked cellular atrophy with architectural disruption).

#### Vestibular endolymphatic Hydrops

A modified four-stage grading system was employed based on saccule-to-utricle size ratio and anatomical relationships (25): Stage 0 (no hydrops: normal saccule and utricle size and position), Stage 1 (mild: slight saccular enlargement without displacement), Stage 2 (moderate: marked saccular enlargement approaching utricle size), and Stage 3 (severe: saccule equal to or larger than utricle with displacement or obliteration of the utriculosaccular junction).

#### Utricular macula evaluation

Pathological changes were assessed according to criteria established by McCall et al. (26), examining for ciliary abnormalities, hair cell loss, basement membrane thickening, stromal edema, and structural disruptions.

### Statistical analysis

Statistical analyses were performed using IBM SPSS Statistics version 23.0 (IBM Corporation, Armonk, NY, United States). Data distribution was assessed using the Shapiro–Wilk test. Categorical variables were analyzed using Pearson’s chi-square test, with Fisher’s exact test applied when expected cell frequencies were less than 5. Continuous variables were compared using the Mann–Whitney U test for non-parametric data or independent samples *t*-test for normally distributed data, as appropriate. Thyroid function parameters were analyzed using paired comparisons within groups (baseline vs. postoperative day 15) and between-group comparisons at each timepoint.

Results are presented as mean ± standard deviation for continuous variables and as frequencies with percentages for categorical variables. For histopathological scoring, both individual section scores and aggregate distributions across experimental groups are reported. Statistical significance was set at *p* < 0.05 for all analyses. Given the exploratory nature of this study and the limited sample size, no adjustments for multiple comparisons were applied. The analyses were hypothesis-driven and focused on predefined anatomical structures known to be involved in endolymphatic hydrops, rather than on data-driven *post hoc* comparisons.

## Results

### Thyroid function assessment

Serum TSH and T4 measurements confirmed successful induction of biochemical hypothyroidism in the thyroidectomy group while demonstrating stable thyroid function in sham and control groups.

*TSH levels*: In the thyroidectomy group, TSH concentrations increased significantly from baseline values of 4.316 ± 0.154 μIU/mL to 4.694 ± 0.195 μIU/mL at postoperative day 15 (mean difference: 0.378 μIU/mL, 95% CI: 0.183–0.573, *p* = 0.021), indicating appropriate compensatory pituitary response to diminished thyroid hormone feedback. In contrast, sham surgery animals showed no significant TSH changes between baseline (3.953 ± 0.357 μIU/mL) and postoperative day 15 (4.063 ± 0.605 μIU/mL, *p* = 0.965), confirming that surgical stress alone did not affect thyroid axis function. Similarly, the control group demonstrated stable TSH levels (baseline: 4.373 ± 0.362 μIU/mL; day 15: 4.354 ± 0.393 μIU/mL, *p* = 0.773). Between-group comparisons at postoperative day 15 revealed significantly higher TSH levels in the thyroidectomy group compared to sham (*p* = 0.029) and control (*p* = 0.029) groups.

*Free T4 levels*: Total thyroidectomy resulted in significant free T4 reduction from preoperative values of 10.036 ± 0.876 pmol/L to 7.679 ± 0.730 pmol/L at postoperative day 15 (mean difference: −2.357 pmol/L, 95% CI: −3.521 to −1.193, *p* = 0.021), confirming successful thyroid hormone depletion. Neither sham surgery (baseline: 11.777 ± 4.092 pmol/L; day 15: 11.486 ± 3.072 pmol/L, *p* = 0.886) nor control animals (baseline: 11.969 ± 2.980 pmol/L; day 15: 11.865 ± 2.508 pmol/L, *p* = 0.773) showed significant free T4 alterations (Table 2). Free T4 levels were significantly lower in the thyroidectomy group compared to both sham (*p* = 0.029) and control (*p* = 0.029) groups. Importantly, all four thyroidectomized animals (100%) developed biochemical hypothyroidism, as confirmed by elevated TSH and decreased free T4 levels relative to their individual baseline values, demonstrating consistent and complete induction of hypothyroidism in this group.

**Table 2:** Serum thyroid hormone (TSH) levels before and after intervention.

Group	TSH (mIU/mL)			Free T4 (pmol/L)		
	Preoperative	Postoperative	*p*	Preoperative	Postoperative	*p*
Thyroidectomy (*n* = 4)	4.316 ± 0.154	4.694 ± 0.195	0.021	10.036 ± 0.876	7.679 ± 0.730	0.021
Sham (*n* = 4)	3.953 ± 0.357	4.063 ± 0.605	0.965	11.777 ± 4.092	11.486 ± 3.072	0.886
Control (*n* = 4)	4.373 ± 0.362	4.354 ± 0.393	0.773	11.969 ± 2.980	11.865 ± 2.508	0.773

These biochemical findings confirmed successful establishment of hypothyroidism in thyroidectomized animals while demonstrating that sham surgery did not induce thyroid dysfunction, validating the experimental model’s specificity.

### Histopathological findings

A minimum of 10 sections from each temporal bone were systematically examined, yielding 40 evaluable sections per experimental group. Histopathological analysis revealed striking and consistent differences between thyroidectomized and control groups across all examined vestibular structures.

#### Reissner’s membrane pathology

Examination revealed statistically significant differences in Reissner’s membrane morphology between experimental groups (*p* < 0.001). Both control and sham surgery groups demonstrated normal Reissner’s membrane architecture in all examined sections (100%, *n* = 40 each), with the membrane maintaining its characteristic thin, two-cell-layer structure positioned in the normal anatomical location separating scala media from scala vestibuli.

**Figure 1:**
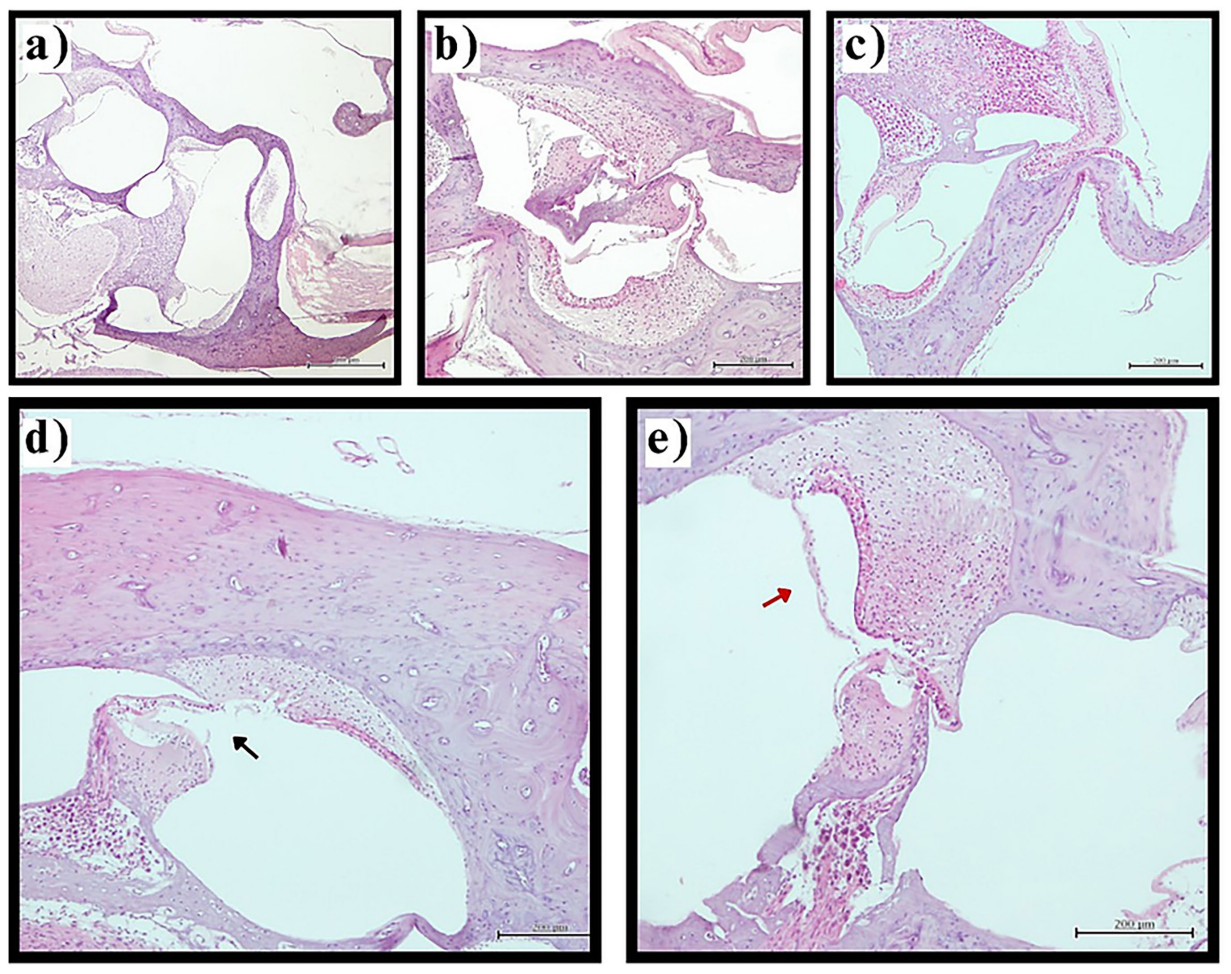
Reissner’s membrane pathology in experimental groups. Representative photomicrographs of cochlear sections demonstrating Reissner’s membrane changes (H&E staining, original magnification ×200). **(a)** Normal Reissner’s membrane architecture in control group showing thin, two-cell-layer structure in physiological position. **(b)** Preserved normal morphology in sham surgery group. **(c)** Mild endolymphatic hydrops in thyroidectomy group with slight displacement of Reissner’s membrane toward scala vestibuli. **(d)** Severe hydrops with membrane rupture (black arrow) in thyroidectomy group demonstrating loss of compartmentalization. **(e)** Membrane thickening (red arrow) in thyroidectomy group indicating pathological cellular changes. Scale bars = 50 μm.

In stark contrast, the thyroidectomy group showed complete absence of normal Reissner’s membrane architecture, with all sections (100%, *n* = 40) demonstrating varying degrees of endolymphatic hydrops. The distribution of hydrops severity was: mild in 40.0% (*n* = 16 sections), moderate in 45.0% (*n* = 18 sections), and severe in 15.0% (*n* = 6 sections) of examined sections. Moderate and severe cases frequently exhibited additional pathological features including membrane thickening, cellular hypertrophy, and in the most severe cases, membrane rupture with loss of compartmentalization between endolymphatic and perilymphatic spaces (Figure 1).

#### Vestibular hydrops

**Figure 2:**
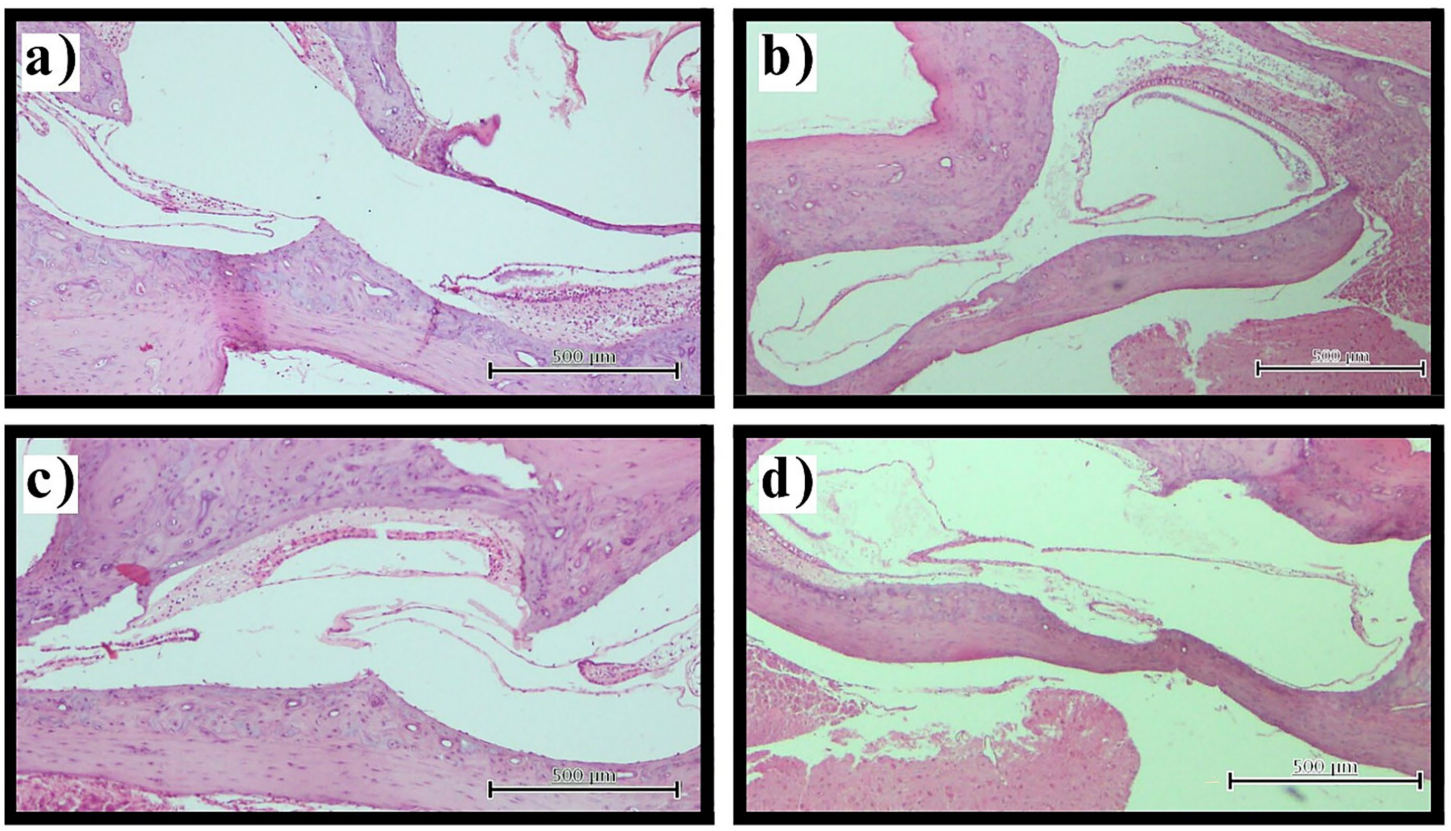
Vestibular hydrops assessment. Representative photomicrographs illustrating vestibular hydrops progression (H&E staining, original magnification ×100). **(a)** Normal vestibular morphology in control group with appropriate saccule and utricle size relationships and intact membranes. **(b,c)** Stage 2 vestibular hydrops in thyroidectomy group showing marked saccular enlargement approaching utricle dimensions. **(d)** Stage 3 vestibular hydrops in thyroidectomy group demonstrating severe saccular distension with displacement of the utriculosaccular junction. Scale bars = 100 μm.

Assessment of the vestibular apparatus demonstrated statistically significant differences between groups (*p* < 0.001). Control and sham surgery groups exhibited normal vestibular morphology (Stage 0) in all examined sections (100%), with saccule and utricle maintaining appropriate size relationships and anatomical positions. The thyroidectomy group showed universal development of advanced vestibular hydrops, with complete absence of normal morphology across all sections. The severity distribution revealed Stage 2 hydrops in 60.0% (*n* = 24) and Stage 3 hydrops in 40.0% (*n* = 16) of sections. Notably, no thyroidectomized specimens exhibited Stage 0 or Stage 1 changes, indicating that when hydrops developed, it consistently progressed to at least moderate severity. Stage 3 cases demonstrated marked saccular distension with displacement or obliteration of the utriculosaccular junction, reflecting severe disruption of normal vestibular anatomy (Figure 2).

#### Stria vascularis pathology

Evaluation of the stria vascularis, the primary structure responsible for endolymph production and ionic homeostasis, demonstrated significant alterations between experimental groups (*p* < 0.001). Control and sham surgery groups maintained normal stria vascularis morphology without evidence of intermediate cell contraction in all examined specimens (100%).

**Figure 3:**
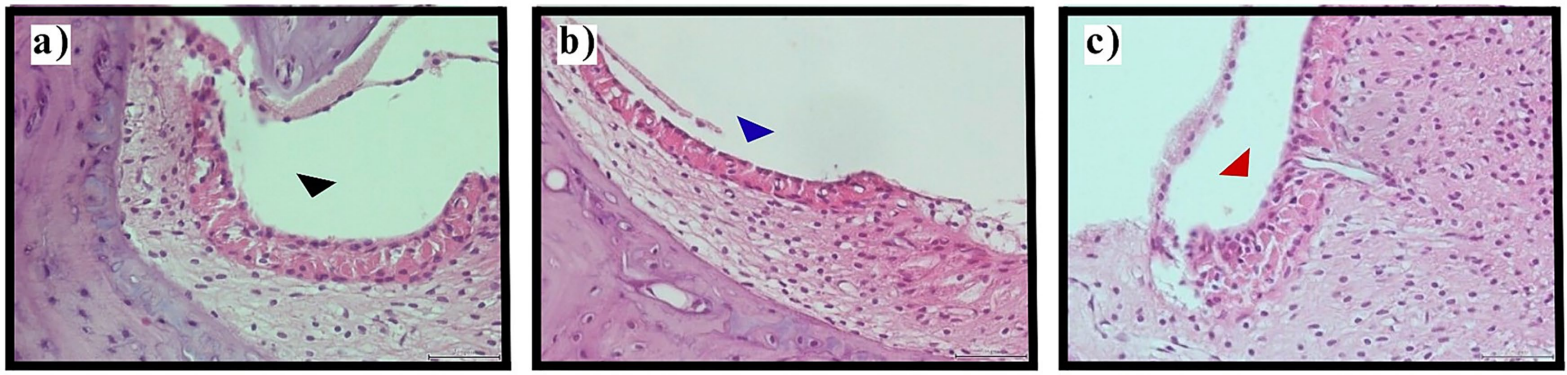
Stria vascularis evaluation. Representative photomicrographs demonstrating stria vascularis pathology (H&E staining, original magnification ×400). **(a)** Normal stria vascularis morphology in control group with preservation of intermediate cell architecture (black arrowhead). **(b)** Mild intermediate cell contraction in thyroidectomy group (blue arrowhead) indicating early pathological changes. **(c)** Moderate intermediate cell contraction in thyroidectomy group (red arrowhead) showing marked cellular shrinkage. Scale bars = 25 μm.

Thyroidectomized animals exhibited universal stria vascularis pathology, with mild intermediate cell contraction observed in 65.0% (*n* = 26) and moderate contraction in 35.0% (*n* = 14) of sections. No sections from thyroidectomized animals showed normal morphology or severe contraction. The consistent presence of intermediate cell contraction, even in its milder forms, suggests fundamental disruption of stria vascularis function and endolymph homeostasis in hypothyroid animals (Figure 3).

#### Utricular macula pathology

Examination of the utricular macula, the sensory epithelium responsible for detecting linear acceleration and head tilt, revealed significant differences between groups (*p* < 0.001). Control and sham surgery groups preserved normal macular architecture in all cases (100%), with intact ciliary bundles, healthy hair cells, normal supporting cell morphology, and preserved basement membrane structure.

**Figure 4:**
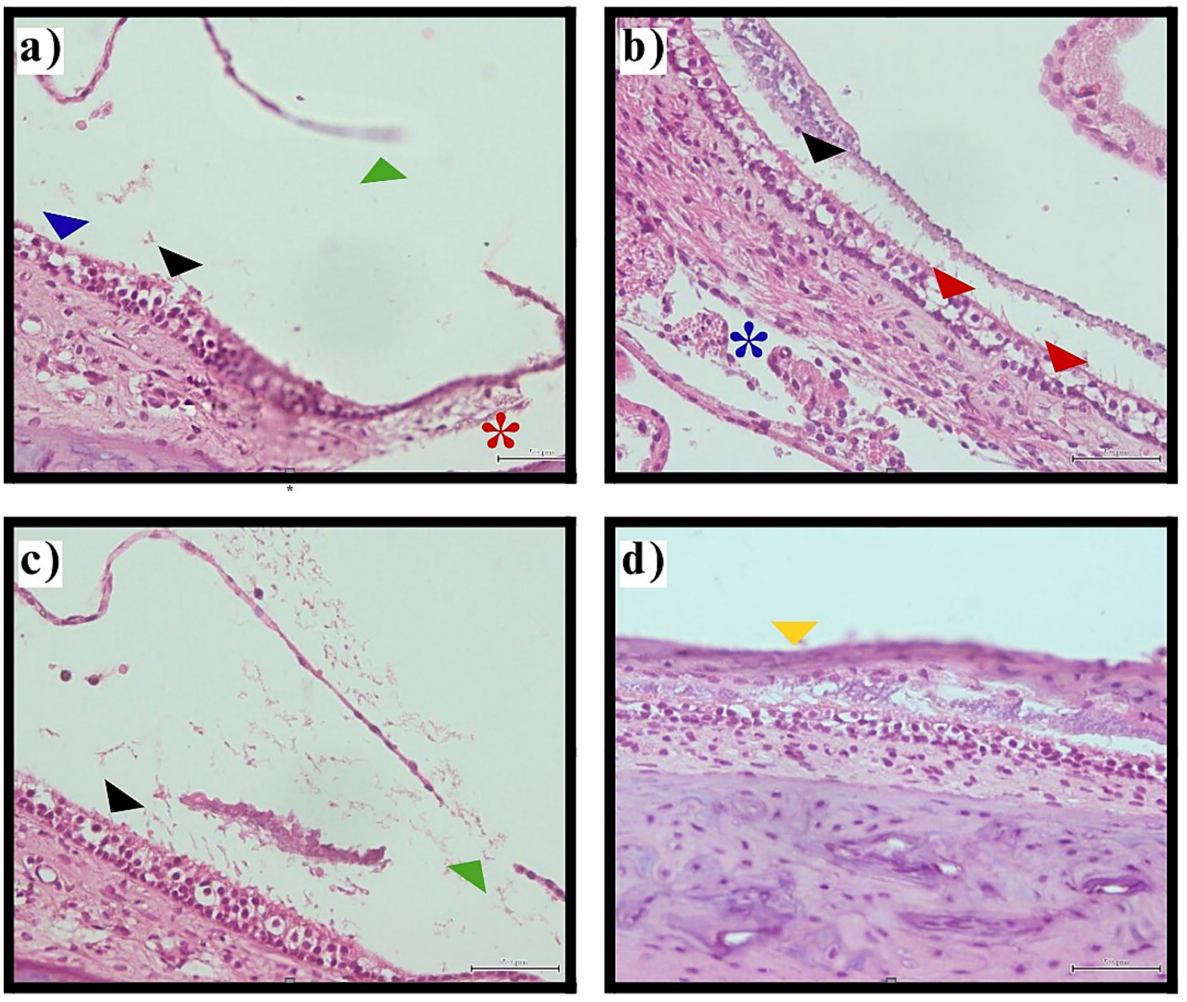
Histological images showing inner ear utricular macula tissue with various annotations. Panel **(a)** highlights structures with blue, black, green, and red markers indicating ciliary abrasion, hair cell loss, and Reissner’s membrane rupture. Panel **(b)** shows tissue with black and red arrows and a blue asterisk indicating ciliary abrasion, decreased hair cell density, and stromal edema. Panel **(c)** contains black and green markers pointing to ciliary disruption and membrane rupture. Panel **(d)** focuses on a section with a yellow marker indicating macular collapse. The images depict pathological changes in the vestibular sensory epithelium following thyroidectomy-induced hypothyroidism. Utricular macula pathology. Representative photomicrographs illustrating utricular macula changes in thyroidectomy group (H&E staining, original magnification ×400).

The thyroidectomy group demonstrated complete macular involvement, with histopathological changes present in 100% (*n* = 40) of examined sections. Observed pathological features included ciliary abrasion and disruption, hair cell loss or degeneration, supporting cell abnormalities, stromal edema, and in some cases, basement membrane pathology. These changes suggest widespread disruption of mechanotransduction capacity in the vestibular sensory epithelium (Table 3, Figure 4).

**Table 3:** Distribution of histopathological findings across experimental groups.

Structure	Grade/stage	Control (*n* = 40)	Sham (*n* = 40)	Thyroidectomy (*n* = 40)	*p*
Reissner’s membrane					<0.001
	Normal	40 (100%)	40 (100%)	0 (0%)	
	Mild hydrops	0 (0%)	0 (0%)	16 (40%)	
	Moderate hydrops	0 (0%)	0 (0%)	18 (45%)	
	Severe hydrops	0 (0%)	0 (0%)	6 (15%)	
Vestibular apparatus					<0.001
	Stage 0	40 (100%)	40 (100%)	0 (0%)	
	Stage 1	0 (0%)	0 (0%)	0 (0%)	
	Stage 2	0 (0%)	0 (0%)	24 (60%)	
	Stage 3	0 (0%)	0 (0%)	16 (40%)	
Stria vascularis					<0.001
	No contraction	40 (100%)	40 (100%)	0 (0%)	
	Mild contraction	0 (0%)	0 (0%)	26 (65%)	
	Moderate contraction	0 (0%)	0 (0%)	14 (35%)	
	Severe contraction	0 (0%)	0 (0%)	0 (0%)	
Utricular macula					<0.001
	Normal	40 (100%)	40 (100%)	0 (0%)	
	Pathological changes	0 (0%)	0 (0%)	40 (100%)	

### Summary of histopathological findings

The histopathological analysis demonstrated universal development of endolymphatic hydrops and associated inner ear pathology in all thyroidectomized animals examined, affecting every measured parameter across all vestibular structures. The 100% penetrance of pathological changes in the thyroidectomy group, contrasted with complete absence of such changes in control and sham surgery groups, establishes a robust association between thyroid hormone deficiency and endolymphatic hydrops development. The consistency of findings across multiple anatomical structures and the systematic nature of pathological changes suggest a fundamental disruption of inner ear homeostasis rather than localized or random pathological processes.

## Discussion

This study provides the first direct experimental evidence that surgically-induced hypothyroidism leads to universal development of endolymphatic hydrops and associated inner ear pathological changes. Our findings demonstrate that thyroidectomized rats developed significant biochemical hypothyroidism, characterized by elevated TSH and decreased T4 levels, accompanied by consistent histopathological evidence of endolymphatic hydrops with varying severity across multiple vestibular structures. The complete absence of these pathological alterations in control and sham-operated groups, combined with 100% penetrance in thyroidectomized animals, strongly suggests a causal relationship between thyroid hormone deficiency and endolymphatic hydrops development.

### Neurological significance of vestibular-thyroid axis

From a neurological perspective, these findings have important implications for understanding the broader impact of thyroid dysfunction on the nervous system. The vestibular system maintains critical connections with multiple central nervous system pathways, including those controlling eye movements through the vestibulo-ocular reflex, postural stability through vestibulospinal pathways, and spatial cognition through vestibulo-thalamic and vestibulo-hippocampal projections (26, 27). Disruption of peripheral vestibular input through endolymphatic hydrops formation may therefore have cascading effects on these interconnected neurological systems.

Recent neuroimaging studies have demonstrated that vestibular disorders can induce structural and functional changes in the cerebral cortex, particularly in regions associated with spatial processing and multisensory integration (28). Our demonstration that hypothyroidism induces vestibular pathology suggests a potential mechanism through which thyroid dysfunction might contribute to neurological symptoms extending beyond the classical manifestations of hypothyroidism, including impaired spatial cognition, increased fall risk, and altered gait patterns.

### Mechanistic relationship to Ménière’s disease

Endolymphatic hydrops is recognized as the fundamental pathological substrate of Ménière’s disease, first demonstrated by Kimura and Schuknecht (8) through their pioneering work showing that endolymphatic duct obstruction in guinea pigs produces hydrops. Subsequent animal studies have confirmed that endolymphatic hydrops development produces Ménière-like symptoms, with symptom severity correlating with hydrops degree (6, 29). Our findings extend this body of work by demonstrating that hypothyroidism represents a potential etiological factor in hydrops formation.

This translational relevance is further supported by recent work highlighting the diagnostic discordance frequently observed in Ménière’s disease. In particular, the study by van Steekelenburg et al. (30) demonstrated that structural endolymphatic hydrops may be present despite normal or inconclusive results on conventional vestibular and audiological tests, underscoring the complex relationship between inner ear pathology and clinical manifestations. These findings align with our histopathological results, suggesting that hypothyroidism-induced hydrops may represent a structural substrate that precedes or escapes detection by standard functional testing, thereby contributing to the clinical diagnostic puzzle of Ménière’s disease.

The gold standard for confirming endolymphatic hydrops in animal models remains histopathological examination after temporal bone sectioning, despite advances in diagnostic modalities including three-dimensional fluid-attenuated inversion recovery magnetic resonance imaging, electrocochleography, cervical vestibular evoked myogenic potentials, and videonystagmography (29). While these techniques provide valuable information about endolymphatic space enlargement and associated physiological changes, direct histological visualization offers definitive confirmation. Our systematic evaluation of multiple inner ear structures using standardized semiquantitative scoring systems provides robust evidence for the relationship between thyroid hormone deficiency and comprehensive inner ear pathology.

### Clinical correlations and implications

Our experimental findings provide mechanistic explanation for clinical observations reported by Santosh and Rao (15), who demonstrated that thyroid hormone levels influence Ménière’s disease symptom severity and that levothyroxine treatment improves symptoms in hypothyroid patients. By histopathologically demonstrating that hypothyroidism causes endolymphatic hydrops, our study establishes the pathophysiological basis for these clinical observations. Since endolymphatic hydrops is the fundamental pathology of Ménière’s disease, these findings prove that thyroid hormone deficiency can lead to Ménière-like symptoms through hydrops formation.

Recent epidemiological evidence has identified hypothyroidism as a tentative risk factor for Ménière’s disease, though with conflicting results across studies (31). Lin et al. (32) demonstrated that hypothyroidism represents an independent risk factor for Ménière’s disease in a large population-based cohort study, while Kim et al. (33) found significant associations between Ménière’s disease and thyroid disorders. However, evidence remained inconclusive due to limited experimental mechanistic studies. Our experimental investigation provides crucial mechanistic evidence supporting these epidemiological observations by demonstrating that hypothyroidism directly induces the pathological hallmark of Ménière’s disease.

Beyond epidemiological associations, recent clinical studies help link structural pathology to symptom expression in Ménière’s disease. Malhoub et al. (34) demonstrated that cochlear and vestibular symptoms may progress independently, while Choi et al. (30) showed that severe endolymphatic hydrops can be present despite normal video head impulse test results, indicating discordance between structural pathology and vestibular function tests. Together, these findings provide a clinical framework for our experimental results, suggesting that hypothyroidism-induced endolymphatic hydrops may underlie heterogeneous and dissociated symptom profiles observed in Ménière’s disease.

The findings also provide mechanistic support for recent clinical observations by Bougerolle et al. (16), who demonstrated significant associations between hypothyroidism and vestibular pathologies in 422 patients. While their study revealed that hypothyroidism increased expression of vestibular instability and gait disorders in Ménière’s disease patients, our experimental model provides the histopathological basis for these observations. The universal development of endolymphatic hydrops in all examined inner ear structures explains why hypothyroid patients experienced more severe vestibular symptoms.

### Subclinical vestibular dysfunction in thyroid disease

Particularly compelling are recent observations by Seymen et al. (17) demonstrating that children with Hashimoto’s thyroiditis exhibited significant vestibular dysfunction on video head impulse test and vestibular evoked myogenic potential testing despite being clinically asymptomatic and biochemically euthyroid. They found that 80.6% of euthyroid children with Hashimoto’s thyroiditis had abnormal vestibulo-ocular reflex gain values, suggesting subclinical vestibulopathy. Our observation of universal endolymphatic hydrops development in all thyroidectomized rats provides a potential histopathological correlate for these electrophysiological findings.

The parallel between electrophysiological abnormalities in asymptomatic patients and our histopathological observations suggests that thyroid dysfunction may induce vestibular pathology before clinical symptoms become apparent. This has important implications for clinical practice, suggesting that patients with thyroid disorders, particularly those with autoimmune thyroiditis, might benefit from vestibular screening even in the absence of overt symptoms.

### Pathophysiological mechanisms

The pathophysiological mechanisms by which thyroid hormones influence inner ear function have been proposed but not experimentally validated until now. Previous studies suggested that thyroid hormones modulate inner ear homeostasis through several mechanisms: regulation of endolymphatic sac function, modulation of ion transport proteins in the stria vascularis, influence on vascular perfusion of inner ear structures, and effects on cochlear and vestibular development (15, 33).

Our histopathological findings provide direct evidence supporting these theoretical mechanisms. The universal development of endolymphatic hydrops across all examined structures confirms disruption of inner ear fluid homeostasis. The observed stria vascularis contraction in all thyroidectomized animals indicates impaired function of this structure, which is primarily responsible for endolymph production and maintenance of the endocochlear potential. Since the stria vascularis contains numerous ion transporters whose expression and function may be thyroid hormone-dependent, its pathological alteration in hypothyroid animals provides a potential mechanistic link between thyroid hormone deficiency and endolymph volume dysregulation.

The involvement of the utricular macula in all thyroidectomized animals, with evidence of hair cell pathology and ciliary disruption, suggests that hypothyroidism affects not only fluid homeostasis but also the mechanosensory apparatus itself. Thyroid hormones are known to influence protein synthesis, cellular metabolism, and mitochondrial function, all of which are critical for maintaining the high metabolic demands of inner ear sensory cells. Hypothyroidism-induced reduction in these cellular processes might compromise the ability of hair cells and supporting cells to maintain normal structure and function.

### Integration with existing literature

Our findings complement and extend existing knowledge about hormonal influences on vestibular function. Previous research has demonstrated that various hormonal factors, including estrogen, progesterone, and aldosterone, can influence inner ear function and contribute to Ménière’s disease pathophysiology (35). The demonstration that thyroid hormones also play a critical role adds another dimension to our understanding of endocrine influences on vestibular homeostasis.

The vestibular system’s sensitivity to hormonal influences may reflect its evolutionary importance for survival, requiring multiple regulatory mechanisms to ensure optimal function across varying physiological states. From a neurological standpoint, the vestibular-endocrine axis represents an important example of how systemic hormonal status can influence sensory function and, consequently, neurological symptoms and quality of life.

### Clinical implications for neurological practice

These findings have several important implications for clinical neurology and neuro-otology practice. First, they support the inclusion of thyroid function assessment in the evaluation of patients presenting with vestibular symptoms, particularly those with Ménière’s disease or unexplained episodic vertigo. Second, they suggest that optimization of thyroid hormone status might represent a therapeutic target in managing vestibular symptoms in hypothyroid patients.

Third, our findings raise the possibility that even subclinical hypothyroidism or autoimmune thyroid disease without overt hormonal dysfunction might contribute to vestibular pathology, warranting consideration of vestibular screening in these populations. Finally, the demonstration that thyroid hormone deficiency can induce structural pathology in the vestibular system underscores the importance of adequate thyroid hormone replacement therapy, not only for metabolic homeostasis but also for maintaining neurological function.

### Study limitations and future directions

This study has several limitations that should be acknowledged. Although sample size was justified using the resource equation approach, the number of animals per group (*n* = 4) was relatively small, which may limit detection of subtle differences and generalizability. However, the 100% penetrance of pathological changes in thyroidectomized animals with complete absence in control groups demonstrates a robust biological effect (effect size: Cohen’s d > 3.0). These findings warrant confirmation in larger cohorts to assess the full spectrum of pathological severity and potential variability. The consistency of findings—affecting every measured parameter across all vestibular structures in thyroidectomized animals while being completely absent in control groups—provides strong evidence despite the limited sample size.

Another limitation of this study is the absence of statistical adjustment for multiple comparisons despite the evaluation of several inner ear structures. However, this study was designed as an exploratory investigation with predefined outcomes based on established histopathological markers of endolymphatic hydrops. Nevertheless, the possibility of inflated type I error cannot be fully excluded, and the findings should be interpreted with this consideration in mind.

The exclusive use of male rats represents another limitation of this study. Ménière’s disease shows female predominance in clinical populations, and sex hormones are known to interact with both thyroid function and inner ear physiology. Estrogen and progesterone influence fluid homeostasis and may modulate the vestibular system’s response to hormonal perturbations. Therefore, future studies should include female animals to determine whether hypothyroidism induces endolymphatic hydrops similarly in both sexes and to explore potential interactions between thyroid hormones and sex steroids in vestibular pathology.

Histopathological changes were evaluated at a single time point (28 days post-thyroidectomy), precluding assessment of the temporal evolution of hydrops development. Earlier or intermediate time points were not examined in this study; therefore, whether hydrops develops progressively or reaches a plateau remains unknown. Future studies employing serial time points (e.g., 7, 14, 21, and 28 days) would help characterize the temporal dynamics of hydrops formation and identify the critical window during which pathological changes first emerge. Such information would be valuable for determining optimal timing for potential therapeutic interventions.

While we demonstrated structural changes associated with hypothyroidism-induced endolymphatic hydrops, we did not assess functional outcomes such as hearing thresholds, vestibular function tests, or behavioral manifestations of vestibular dysfunction. Future investigations should incorporate auditory brainstem response testing, vestibulo-ocular reflex measurements, and behavioral assessment of balance and spatial orientation to establish functional correlates of the observed histopathological changes.

Additionally, this study did not investigate the reversibility of endolymphatic hydrops with thyroid hormone replacement therapy. This represents a critical question with important therapeutic implications. If hydrops proves reversible with early hormone replacement, it would support aggressive treatment of hypothyroidism in patients with vestibular symptoms. Conversely, if structural changes prove irreversible beyond a certain point, it would emphasize the importance of early detection and prevention.

It should also be noted that calcium chloride supplementation was administered exclusively to the thyroidectomy group to prevent hypocalcemic complications. Although this represents a methodological difference between groups, we consider it unlikely to confound our findings. Calcium supplementation would be expected to exert protective rather than detrimental effects on inner ear structures, as calcium homeostasis is critical for hair cell mechanotransduction and synaptic function. Furthermore, untreated hypocalcemia can itself induce vestibular symptoms, potentially confounding the assessment of hypothyroidism-specific effects. Therefore, the direction of any calcium-related effect would oppose rather than explain the observed vestibular pathology. Nevertheless, future studies could include a calcium-supplemented sham group to definitively exclude this variable.

Future research should also explore the molecular mechanisms underlying thyroid hormone effects on inner ear structures, including identification of thyroid hormone receptors in specific inner ear cell types, characterization of thyroid hormone-responsive genes in the stria vascularis and vestibular sensory epithelia, and investigation of downstream signaling pathways. Understanding these mechanisms could identify novel therapeutic targets independent of thyroid hormone replacement.

## Conclusion

This study provides the first direct experimental evidence that hypothyroidism induces endolymphatic hydrops, the fundamental pathological hallmark of Ménière’s disease. Thyroidectomy-induced hypothyroidism led to universal development of endolymphatic hydrops across all examined inner ear structures, including Reissner’s membrane, vestibular apparatus, stria vascularis, and utricular macula. These histopathological changes provide a mechanistic explanation for clinical associations between hypothyroidism and Ménière’s disease reported in previous epidemiological studies.

From a neurological perspective, these findings establish the thyroid-vestibular axis as an important consideration in evaluating and managing patients with vestibular disorders. The results support the hypothesis that thyroid hormones play a critical role in maintaining inner ear fluid homeostasis and vestibular function, and suggest that thyroid dysfunction should be systematically assessed in patients presenting with vestibular symptoms. Further research investigating the reversibility of these changes with thyroid hormone replacement therapy, the molecular mechanisms underlying thyroid hormone effects on inner ear structures, and the functional consequences of hypothyroidism-induced vestibular pathology may open new therapeutic avenues for managing vestibular disorders associated with hypothyroidism and enhance our understanding of neuroendocrine influences on the vestibular system.

## Data Availability

The original contributions presented in the study are included in the article/supplementary material, further inquiries can be directed to the corresponding author.
